# Metastatic Colonic Adenocarcinoma in Breast: Report of Two Cases and Review of the Literature

**DOI:** 10.1155/2015/458423

**Published:** 2015-03-25

**Authors:** Jiten P. Kothadia, Rezina Arju, Monica Kaminski, Arvind Ankireddypalli, Sushil Duddempudi, Jonathan Chow, Shah Giashuddin

**Affiliations:** ^1^Department of Internal Medicine, The Brooklyn Hospital Center, An Academic Affiliate of Icahn School of Medicine at Mount Sinai, 121 DeKalb Avenue, Brooklyn, NY 11201, USA; ^2^Department of Family Medicine, The Brooklyn Hospital Center, An Academic Affiliate of Icahn School of Medicine at Mount Sinai, 121 DeKalb Avenue, Brooklyn, NY 11201, USA; ^3^Department of Gastroenterology, The Brooklyn Hospital Center, An Academic Affiliate of Icahn School of Medicine at Mount Sinai, 121 DeKalb Avenue, Brooklyn, NY 11201, USA; ^4^Department of Pathology, The Brooklyn Hospital Center, An Academic Affiliate of Icahn School of Medicine at Mount Sinai, 121 DeKalb Avenue, Brooklyn, NY 11201, USA

## Abstract

Metastatic adenocarcinoma to the breast from an extramammary site is extremely rare. In the literature, the most current estimate is that extramammary metastases account for only 0.43% of all breast malignancies and that, of these extramammary sites, colon cancer metastases form a very small subset. Most commonly seen metastasis in breast is from a contralateral breast carcinoma, followed by metastasis from hematopoietic neoplasms, malignant melanoma, sarcoma, lung, prostate, and ovary and gastric neoplasms. Here we present two rare cases, in which colonic adenocarcinomas were found to metastasize to the breast. In both cases, core biopsies were obtained from the suspicious areas identified on mammogram. Histopathology revealed neoplastic proliferation of atypical glandular components within benign breast parenchyma which were morphologically consistent with metastatic adenocarcinoma. By immunohistochemical staining, it was confirmed that the neoplastic components were immunoreactive to colonic markers and nonreactive to breast markers, thus further supporting the morphologic findings. It is extremely important to make this distinction between primary breast cancer and a metastatic process, in order to provide the most effective and appropriate treatment for the patient and to avoid any harmful or unnecessary surgical procedures.

## 1. Introduction

Colorectal cancer is the third most common type of cancer globally, and most frequently it spreads to the regional lymph nodes, liver, lungs, and bone, in descending order [1]. It is very rare to find metastasis of colonic adenocarcinoma to the breast and, thus, they often may be confused with primary neoplasms of the breast [2]. The median age at which these metastases occur is 54 years and the prognosis is poor because they indicate an advanced or disseminated disease [1, 3]. The first described case of colon cancer metastasizing to the breast was reported in 1974 by McIntosh and, since that time, there are only a few reported cases in the literature [4].

Obtaining a detailed past medical history, appropriate radiologic work-up, followed by a tissue biopsy with histologic and immunohistochemical work-up, has proven to be extremely helpful in determining the source of the malignancy. Anti-cytokeratin 7 (CK 7) and anti-cytokeratin 20 (CK 20) antibodies have been used to differentiate between cancers of a primary or metastatic source [2]. CK 7 (−)/CK 20 (+) represents a colonic adenocarcinoma staining pattern, whereas CK 7 (+)/CK 20 (−) represents a breast carcinoma staining pattern [2]. The use of additional breast tumor markers, such as estrogen receptor (ER), progesterone receptor (PR), and human epidermal growth factor receptor 2 (Her2/neu), can be used for confirmation of the source of the tumor [2]. It is necessary to identify the source of the tumor accurately, in order to determine the most appropriate line of treatment for the patient and, indeed, to avoid any unnecessary radical surgery [5, 6]. Here we have described two cases of adenocarcinoma of colorectal origin that spread to the unilateral breast with distinguishing histologic and immunohistochemical features between primary breast malignancies and metastatic adenocarcinoma of colorectal origin.

## 2. Case Report

### 2.1. Case 1

**Figure 1:**
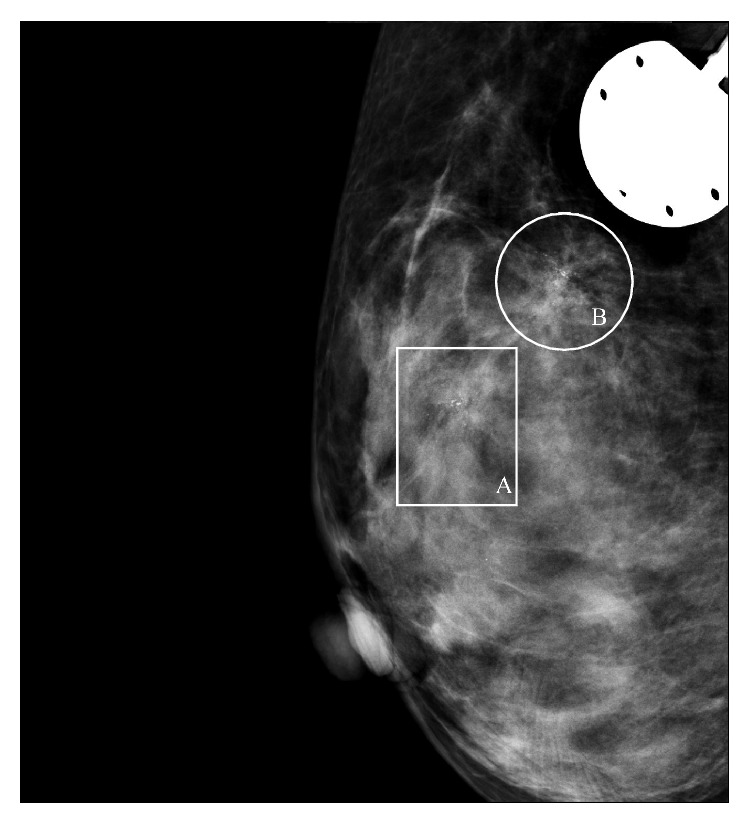
Mammographic image of the breast showing 2 groupings (A and B) of microcalcifications in the upper medial aspect of the right breast.

A 45-year-old woman with history of stage 4 colon cancer, status after right hemicolectomy in 2012, presented to the outpatient clinic for a follow-up visit and biannual work-up. The patient had received Oxaliplatin with 5-fluorouracil and folinic acid (FOLFOX) and Avastin (bevacizumab) chemotherapy following surgery. Although the CT scans of her lungs and liver did not show any evidence of metastatic disease, the mammogram showed an abnormal density associated with two clusters of microcalcifications in the upper medial aspect of the right breast. The anterior cluster measured approximately 6.8 mm × 5.3 mm in size and was located 3.64 cm from the nipple (Figure 1). The posterior cluster measured 9.3 mm × 7.4 mm and was located 6-7 cm from the nipple. Both clusters showed pleomorphic appearance with linear distribution which was indeterminate but suspicious for neoplasm (BI-RADS 4).

The patient was subsequently sent for a stereotactic guided core biopsy of two areas of these microcalcifications. The procedure was carried out using a petite biopsy needle and a Suros EVIVA vacuum to obtain core samples. The core biopsy samples were radiographed with iCAD 7.2, which confirmed the presence of microcalcifications in the samples. Surgical microclips under stereotactic guidance were placed at the biopsy sites.

The morphology of all four core biopsy samples revealed benign appearing mammary tissue, interspersed by proliferation of atypical glandular elements, morphologically consistent with adenocarcinoma. A few of the glands showed intraductal microcalcifications, compatible with prior mammogram. In some glands, microcalcifications were accompanied by necrotic cellular debris. A few of the normal appearing breast ducts also showed intraductal microcalcifications. Nuclei of these malignant appearing cells showed hyperchromasia, nuclear crowding with pseudostratification. Considering the prior history of colonic adenocarcinoma, additional immunohistochemical markers were tested, besides the usual hormone receptor studies. The neoplastic glands that are associated with microcalcifications and necrotic cellular debris were positive for CDX2 and CK 20 and negative for CD7, ER, PR, and Her2-Neu. In routine histology, these atypical glands were not surrounded by a convincing myoepithelial layer, as shown by the negative markers for calponin, CD10, and p63. Thus, the diagnosis of metastatic colonic adenocarcinoma was confirmed. The patient was discharged home with outpatient appointments with medical oncologist for further management.

### 2.2. Case 2

A 56-year-old female with history of stage 4 colonic adenocarcinoma with metastasis to the liver, status after resection in 2009, presented to the emergency department with complaints of nausea, vomiting, and abdominal pain. The patient had received radiotherapy and completed six cycles of chemotherapy consisting of Oxaliplatin, 5-fluorouracil and folinic acid (FOLFOX), and Avastin (bevacizumab). The last cycle of chemotherapy was seven weeks prior to presentation.

**Figure 2:**
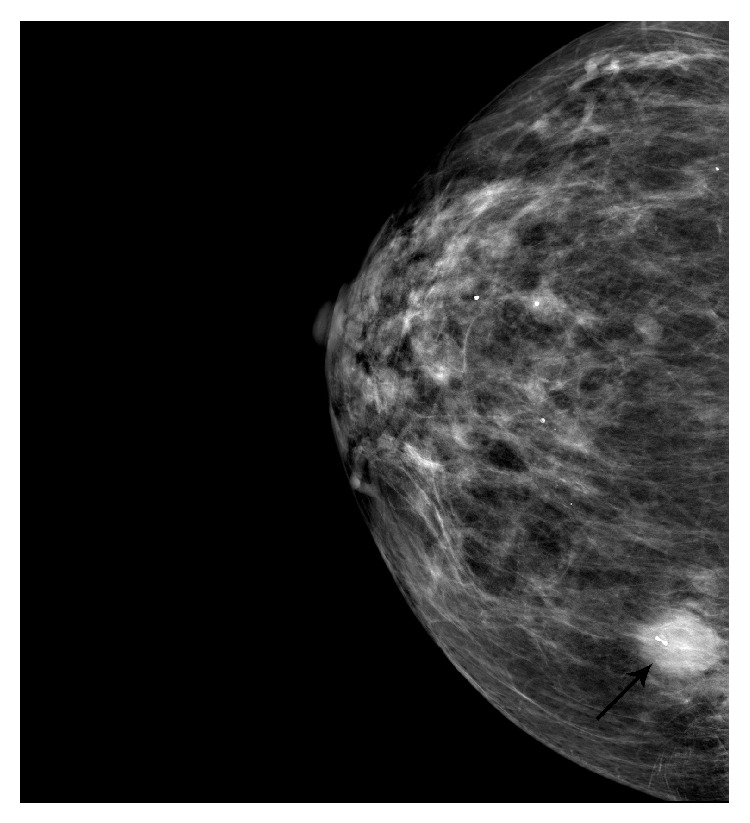
Mammographic image of the breast showing highly vascular spiculated hypoechoic mass measuring 1.35 cm × 1.46 cm × 1.22 cm (arrow).

In the emergency room, during the physical examination, the patient stated that she noticed a lump in her right breast. On palpation, a small mass was felt deep in the inferior medial aspect of the right breast at 4 o'clock and 9.7 cm from the nipple. No discharge was noted from the nipple. A bilateral digital diagnostic mammogram study was obtained with views in the craniocaudal and mediolateral oblique positions and with additional spot compression views due to the deep nature of the mass. It was found to be highly vascular, speculated, and hypoechoic mass measuring 1.35 cm × 1.46 cm × 1.22 cm (Figure 2). Some benign appearing microcalcifications were also noted bilaterally, but there were no other masses seen in either breast. Additionally, no axillary lymphadenopathy, skin thickening, or nipple retraction was noted.

**Figure 3:**
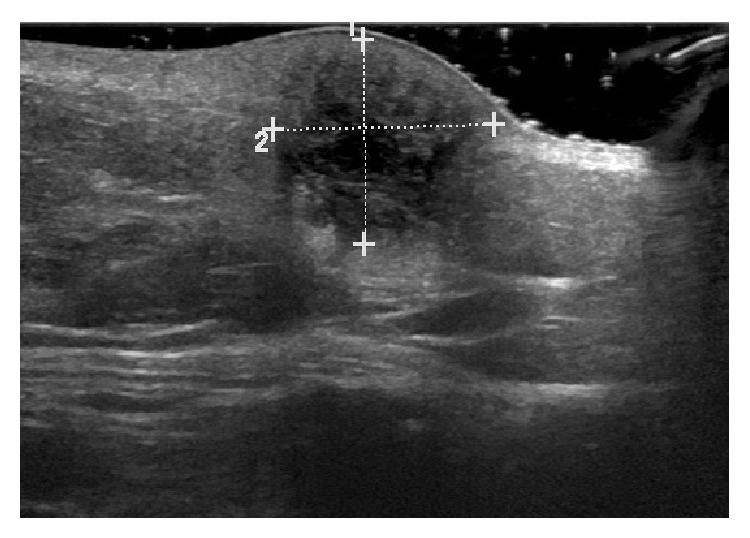
Ultrasonography image of the right breast showing 1.4 cm × 1.2 cm irregularly shaped, speculated, hypoechoic lesion with central necrotic changes and high vascularity.

A right breast ultrasound was performed at the same time and showed the palpable mass to be an irregularly shaped, spiculated, hypoechoic lesion with central necrotic changes and high vascularity (Figure 3). No lymph nodes were seen in the right axilla. The patient was sent for an ultrasound guided core biopsy of the lesion. Histopathologic examination of the core biopsy samples showed atypical glandular proliferation within benign appearing breast parenchyma. Cells in these atypical glands showed enlarged, hyperchromatic nuclei, prominent nucleoli with nuclear crowding and pseudostratification. There was no evidence of in situ ductal or lobular carcinoma noted in these biopsy samples. Atypical glands and a few benign breast ducts were associated with microcalcifications and scant, intraglandular necrotic debris. All these findings were morphologically consistent with a diagnosis of adenocarcinoma. Upon further work-up by immunohistochemical staining, the neoplastic glands were shown to be positive for CDX2 and CK 20 (focal), while being negative for CK 7, ER, PR, Mammaglobin, and GCDFP-15. Overall, the tumor was morphologically and immunophenotypically consistent with a metastatic adenocarcinoma of colorectal origin. The diagnosis was further confirmed by a series of discussions with the colorectal surgeon and medical oncologist who had treated the patient earlier. Given the patient's advanced metastatic disease and her poor functional status it was decided that chemotherapy would cause more harm than good. The patient was subsequently referred to a palliative care team and the patient decided to go for home hospice care.

## 3. Discussion

Colorectal carcinoma is the third most common cancer among men and women with an estimated number of 71,830 reported new cases in men and 65,000 new cases in women [7]. The most common sites of spread include locoregional lymph nodes, liver, lungs, and bone in descending order [1]. It is rare to find metastasis of colonic adenocarcinoma to the breast [5, 6]. The incidence of metastasis in breast from extramammary sites was 6.6% to 7% in autopsy studies [8].

The most common tumor that metastasizes to the breast is a contralateral breast carcinoma, followed by metastasis from hematopoietic neoplasms, malignant melanoma, lymphoma, sarcoma, lung, prostate, ovary, kidney, stomach, and carcinoid tumors [1, 3–5, 9]. In the published literature, there are very few reported cases of colorectal cancer that metastasized to the breast, and these are mainly seen in the settings of concomitant liver and lung metastasis [9]. The median age at which breast metastasis present is 54 years, and it is much more commonly found in women than in men (5–8%) [5, 6, 8]. The average time interval to develop metastatic lesions in the breast from a primary source is about two years [5]. These findings were consistent with the patients presented in this report as they were women aged 45 and 56, respectively.

The commonly known explanation for metastasis involves the spread of cells from the primary tumor via lymphatic, hematogenous, or transcoelomic spread. This model does not explain the finding of a metastatic lesion in the breast as the only site of spread [10]. A second hypothesis was proposed by Mihai et al., suggesting that when cancer cells undergo apoptosis, small fragments of genome may be released, enter circulation, enter other cells of the reticuloendothelial system, and maybe even enter normal cells via the route of transinfection [10].

Morphologically, metastases to the breast tend to show certain characteristics, including a periductal and perilobular location, lack of an in situ ductal or lobular component, and the absence of a desmoplastic reaction [6, 11]. They tend to have rapid growth and be palpable mobile masses and are not associated with any skin dimpling, nipple retraction, or bloody nipple discharge [1, 6, 11]. They are slightly more common in the left breast and tend to be found in the upper outer quadrant [4]. Neither patient presented in this report was found to have any nipple discharge or retraction. Case 2 presented with a palpable mass in one breast, although Case 1 did not have any masses present at all.

The imaging studies to evaluate metastatic breast lesions include ultrasonogram and mammography. On ultrasound examination of metastatic breast lesions, hypoechoic nodules with indistinct and irregular margins are often seen [6]. Penetrating vascularity may or may not be found, but the presence of this finding is very suggestive for malignancy [6]. Case 2 was found to have a hypoechoic, irregular, and highly spiculated nodule on the ultrasound of her breast that was highly vascular. This finding is very consistent with the description of metastatic lesions to the breast.

A mammographic finding of metastatic lesions typically shows well-circumscribed lesions without spiculation or thickening of the skin [1, 9]. According to the literature, on mammography, metastasis from the colon is not expected to show microcalcifications [6, 9]. In fact, it is only with metastatic ovarian carcinoma with psammoma bodies that calcifications are commonly found and they are usually not associated with metastases from any other source [1, 4, 5]. Both of the cases presented in this report had unusual mammographic results that showed microcalcifications. Case 1 had two areas of suspicious microcalcifications unilaterally, and in Case 2 there were bilateral microcalcifications observed. Although microcalcifications are not an expected finding, there is a case described by de Bobadilla et al. in which a patient was also found to have microcalcifications on mammography [4]. Thus, microcalcifications are more commonly seen in primary breast tumors and found rarely in tumors other than breast origin and, generally, they do not exclude the possibility of metastasis.

**Figure 4:**
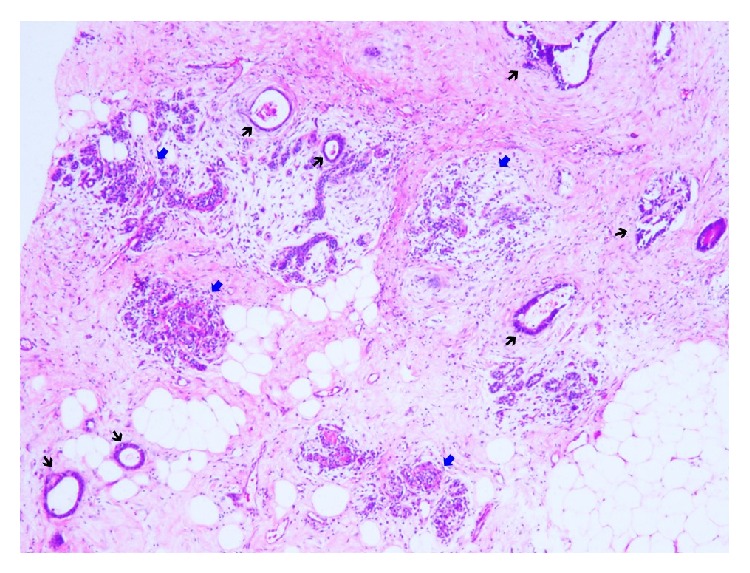
Tissue core biopsy from suspicious areas shows benign appearing breast terminal ductal-lobular units (TDLU) (blue arrows), interspersed by neoplastic colonic glands (black arrows) without any in situ carcinoma component (Hematoxylin and Eosin stain, 40x magnification).

Since metastatic breast lesions can resemble benign or primary breast neoplasm in clinical examination and imaging studies, distinguishing between primary and metastatic breast lesion is not always straightforward. It is most important to reach an accurate diagnosis in order to guide the surgeons and oncologists to provide an appropriate treatment plan for the patient and to avoid any unnecessary radical surgery [1]. The standard of care is to evaluate the tissue sample through image-guided percutaneous biopsy from these lesions; core biopsy is comparatively better than fine needle aspiration biopsy due to the absence of tissue architecture in the latter and less diagnostic sensitivity and specificity comparing to core biopsy [6]. Histologic features that are more consistent with metastatic lesions include a lack of elastosis due to their fast growth, a sharp transition at the border of the tumor, and presence of the tumor in the subcutaneous tissue [5]. Also, the finding of in situ carcinoma is more supportive of a primary breast tumor [5], as opposed to a metastatic process (Figure 4).

**Figure 5:**
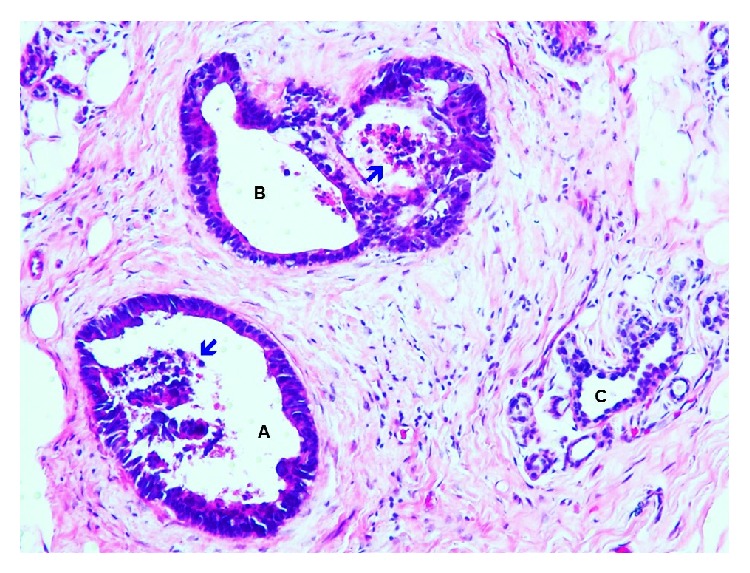
Neoplastic colonic glands (A and B) show hyperchromatic, crowded nuclei, loss of nuclear polarity, and pseudostratification, intermixed with normal breast ducts (C). Necrosis is noted in neoplastic colonic glands (arrow) (Hematoxylin and Eosin stain, 200x magnification).

**Figure 6:**
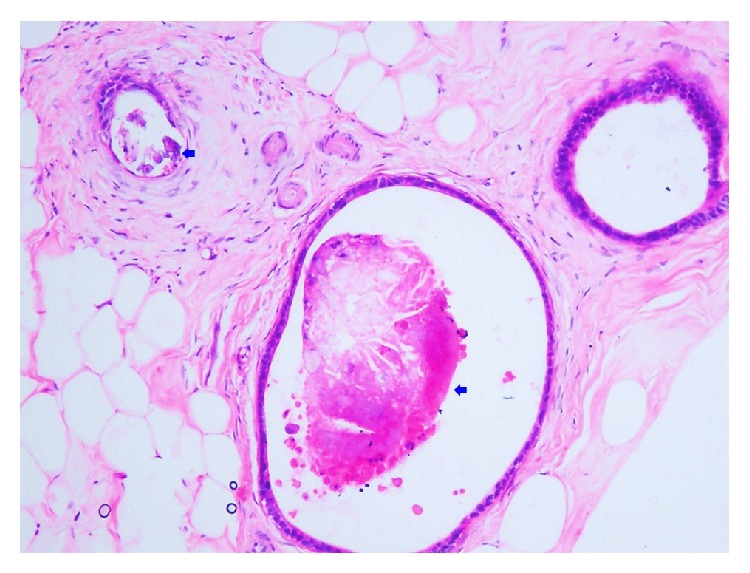
Metastatic colonic glands show intraglandular microcalcifications (arrow) (Hematoxylin and Eosin stain, 200x magnification).

Proliferation of well formed, dilated, and larger sized glands or complex glandular architecture with uniform, basally oriented nuclei and loss of nuclear polarity are commonly seen histologic features that could be observed in both primary ductal adenocarcinoma and metastatic adenocarcinoma of colorectal origin. In both cases presented in this report, on Hematoxylin and Eosin stain, there were proliferation of cuboidal to columnar shaped cells with nuclear hyperchromasia, pseudostratification, and prominent nucleoli. In the majority of the cases, a constellation of nuclear hyperchromasia, pseudostratification, and intraglandular tumor necrosis are more suggestive of colorectal origin (Figure 5) [11]. Our cases show somewhat similar histomorphologic features to those described above and are not readily compatible with a primary breast tumor. Mucinous differentiations, microcalcifications, and the presence of intraglandular tumor necrosis are a few histologic features shared by both primary breast ductal adenocarcinomas and metastatic carcinoma in the breast of colorectal origin. Although rarely seen in the event of colorectal carcinomas, microcalcifications are more commonly associated with primary ductal adenocarcinoma of breast, as seen in our cases (Figure 6).

**Figure 7:**
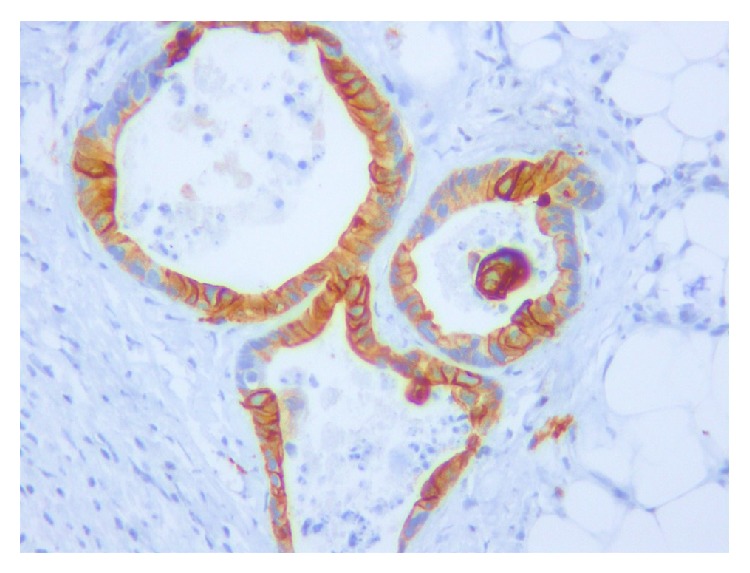
Cytokeratin 20 immunostaining in both cases shows positive cytoplasmic staining in metastatic colonic glands (200x magnification).

**Figure 8:**
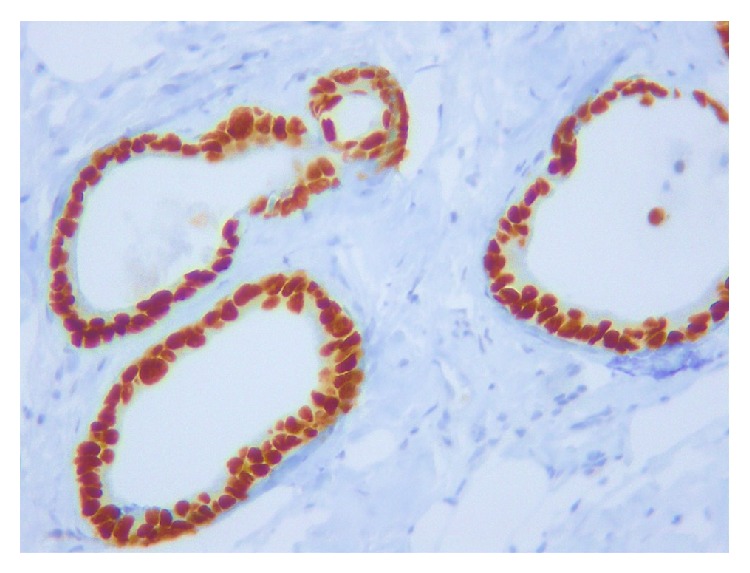
CDX-2 immunostaining in both cases shows intense positivity in nucleus of neoplastic colonic glands (200x magnification).

**Figure 9:**
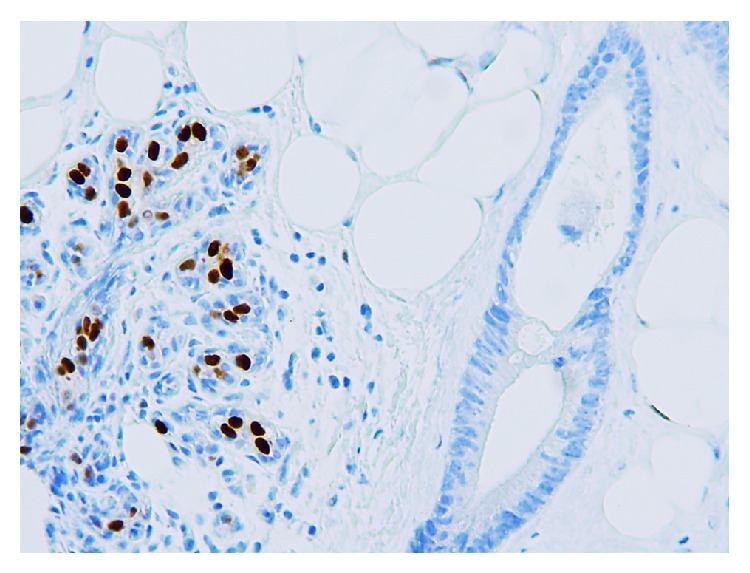
In both of these cases, benign breast ductal cells are immunoreactive to estrogen receptor (left) and nonreactive to metastatic colonic glands (right) (200x magnification).

Further confirmation of the above morphologic findings by immunohistochemical (IHC) staining is always recommended to establish an accurate final diagnosis. Cytokeratin 7 (CK 7) and cytokeratin 20 (CK 20) are the most widely used initial IHC panel [1, 5, 6, 12]. Additionally, positive immunostaining for CDX2 is a highly specific and sensitive marker for colon carcinoma [2, 11]. Most of the primary ductal adenocarcinomas of breast are immunoreactive to cytokeratin 7 (CK 7 (+)) and nonreactive to cytokeratin 20 (CK 20 (−)). Most colorectal carcinomas, unlike ductal adenocarcinoma of the breast, are positive for CK 20 (CK 20 (+)) (Figure 7) and negative for CK 7 (CK 7 (−)) [1, 6, 12]. Additionally, source-specific antibodies can be used to strengthen a diagnosis of colorectal origin, including positive CEA and CDX2 (Figure 8) and negative hormone receptor studies (estrogen receptor, progesterone receptor, and Her-2/Neu) as well, to rule out primary breast origin [12]. In these two cases, both the patients were positive for CXD2 and CK 20 and negative for CK 7, ER, and PR (Figure 9). This is consistent with an adenocarcinoma of colorectal origin and can be used to firmly establish a diagnosis.

The management of patients with metastatic breast cancer is extremely complex and depends upon multiple factors such as age, functional status, comorbidities, distant metastasis, and lymph node status [8]. Since finding an extramammary tumor in the breast represents a highly aggressive disseminated disease, there is little role for surgery unless otherwise indicated in cases with local disease involving the skin, areola, or nipple [13]. Instead, systemic therapy will be required for most of these patients [1, 6, 13]. One study found that when antiepidermal growth factor receptor monoclonal therapy (Cetuximab) was used in combination with standard systemic chemotherapy, there was a higher response rate [6]. Using a combination of local therapy with the systemic chemotherapy may be considered, if the patient has ulceration of the breast mass or invasion of the chest wall, as well as disseminated metastases [6]. A simple mastectomy may be the treatment of choice if the tumor is found to be large in size or located deeply or if it causes severe pain [4]. This decision should be made with extreme caution, as surgery has been shown to carry the risk of seeding the colonic adenocarcinoma to the skin [14].

Unfortunately, the presence of metastatic tumor in the breast carries a dismal prognosis, since it is usually indicative of disseminated disease [1, 4, 6]. The average survival time is estimated to be less than one year from the time of diagnosis of the breast metastasis [1, 3, 6]. Patients with good response to chemotherapy were able to prolong the survival [4].

## 4. Conclusion

Metastatic colonic adenocarcinoma to the breast is an extremely rare clinical entity [5, 6]. Every physician who is involved in a clinical oncologic practice should be extremely cautious in dealing with a breast lesion, as seen in these two cases, since not all breast tumors are of primary breast origin. Besides exploring the detailed clinical background, an appropriate radiologic evaluation is extremely important in the initial triage of any breast lesion and also helps identify any occult malignancy. Evaluation of biopsy material by precise histologic and immunohistochemical studies is always the gold standard to establish a definitive diagnosis of primary versus metastatic tumor of breast and, thus, provide guidance to the surgeons and oncologists to determine the appropriate further management options [5, 6].
